# Modelling the global economic costs of tobacco product waste

**DOI:** 10.2471/BLT.22.288344

**Published:** 2022-08-22

**Authors:** Juleen Lam, John Schneider, Ron Shadbegian, Frank Pega, Simone St Claire, Thomas E Novotny

**Affiliations:** a Department of Public Health, California State University, East Bay, 25800 Carlos Bee Blvd, Hayward, CA, United States of America (USA).; b Avalon Health Economics, Morristown, USA.; c Department of Economics, San Diego State University, San Diego, USA.; d Department of Environment, Climate Change and Health, World Health Organization, Geneva, Switzerland.; e School of Public Health, San Diego State University, San Diego, USA.

## Abstract

Tobacco smoking continues to cause considerable premature mortality and morbidity worldwide. Most of the approximately six trillion cigarettes sold globally each year are discarded improperly as toxic environmental waste. Tobacco product waste, including cigarette butts, is the most commonly collected waste item worldwide. Of particular concern is the cellulose acetate filter, a poorly degradable plastic additive attached to most commercially manufactured cigarettes. This filter was introduced by the tobacco industry to reduce smokers’ perception of harm and risk but it has no health benefit. To inform health policy and practice and improve public health outcomes, governments and society can benefit from cost estimates of preventing, properly disposing of and/or cleaning up tobacco product waste. Estimating the costs of tobacco product waste to communities and responsible authorities could encourage the development of health, environmental and fiscal policy interventions and shift accountability for the costs of tobacco product waste onto the global tobacco industry. To support health and environmental policy-making, we therefore propose an empirical approach to estimate the economic costs of tobacco product waste based on its negative environmental externalities. We first present general estimates for six representative countries and then identify data gaps that need to be addressed to develop global estimates. Interventions against tobacco product waste may be new channels to regulate tobacco products across sectors – for example, health, environment and finance – and consequently reduce overall tobacco use.

## Résumé

Le tabagisme continue à entraîner un taux de morbidité et de mortalité précoce considérable à travers le monde. La plupart des quelque six billions de cigarettes vendues chaque année à l'échelle planétaire ne sont pas correctement éliminées et deviennent une source de pollution environnementale toxique. Les déchets liés aux produits du tabac, notamment les mégots, sont les résidus les plus fréquemment collectés dans le monde. C'est surtout le filtre qui pose problème car il est composé d'acétate de cellulose, un additif plastique difficilement biodégradable que l'on retrouve dans la majorité des cigarettes commercialisées. Ce filtre a été introduit par l'industrie du tabac afin de donner aux fumeurs l'impression qu'ils courent moins de risques, alors qu'il n'a aucun effet bénéfique sur la santé. Les gouvernements et la société pourraient récolter les fruits d'une estimation des coûts engendrés par la prévention, l'élimination correcte et/ou le nettoyage des déchets liés aux produits du tabac, qui leur permettrait de mieux orienter les politiques et pratiques en la matière, mais aussi d'améliorer les résultats de santé publique. Estimer l'impact de ces déchets sur les communautés et les autorités compétentes pourrait encourager à adopter des mesures sanitaires, environnementales et fiscales, et pousser à responsabiliser davantage l'industrie mondiale du tabac vis-à-vis des coûts qu'ils entraînent. En vue de soutenir l'élaboration de politiques sanitaires et environnementales, nous proposons donc une approche empirique visant à déterminer les conséquences économiques des déchets générés par les produits du tabac en nous fondant sur l'influence néfaste qu'ils exercent sur l'environnement. Nous commençons par présenter des estimations globales pour six pays représentatifs, puis nous identifions les lacunes à combler dans les données afin de produire des estimations mondiales. Prendre des mesures de lutte contre ce type de déchets pourrait constituer un nouveau moyen de réglementer les produits du tabac dans différents secteurs comme la santé, l'environnement et les finances par exemple – et, par conséquent, faire diminuer la consommation de tabac en général.

## Resumen

El tabaquismo sigue causando una considerable tasa de mortalidad y morbilidad prematura en todo el mundo. La mayor parte de los casi seis billones de cigarrillos que se venden cada año en el mundo se desechan de forma inadecuada como residuos tóxicos para el medio ambiente. Los residuos de los productos del tabaco, incluidas las colillas, son los que más se recogen en todo el mundo. Un aspecto especialmente preocupante es el filtro de acetato de celulosa, un aditivo plástico poco degradable que se adhiere a la mayoría de los cigarrillos fabricados en el mercado. La industria del tabaco introdujo este filtro para reducir la percepción de daño y riesgo de los fumadores, pero no tiene ningún beneficio para la salud. A fin de fundamentar las políticas y prácticas sanitarias y mejorar los resultados en materia de salud pública, los gobiernos y la sociedad se pueden beneficiar de las estimaciones de costes de la prevención, la eliminación adecuada o la limpieza de los residuos de productos del tabaco. La estimación de los costes de los residuos de productos del tabaco para las comunidades y las autoridades responsables podría fomentar el desarrollo de intervenciones de política sanitaria, medioambiental y fiscal y trasladar la responsabilidad de los costes de los residuos de productos del tabaco a la industria del tabaco mundial. Para apoyar la elaboración de políticas sanitarias y medioambientales, se propone un enfoque empírico para estimar los costes económicos de los residuos de los productos del tabaco en función de sus consecuencias negativas para el medio ambiente. En primer lugar, se presentan estimaciones generales para seis países representativos y, a continuación, se identifican las deficiencias de información que se deben abordar para desarrollar estimaciones globales. Las intervenciones contra los residuos de productos del tabaco pueden constituir canales nuevos para regular los productos del tabaco en todos los sectores, por ejemplo, la salud, el medio ambiente y las finanzas, y, en consecuencia, reducir el consumo general de tabaco.

## ملخص

ما زال تدخين التبغ يتسبب في نسبة كبيرة للوفيات والإصابة بالأمراض في جميع أنحاء العالم. يتم التخلص من معظم ما يقرب من ستة تريليونات سيجارة تُباع عالميًا كل عام على أنها نفايات بيئية سامة. إن نفايات منتجات التبغ، بما في ذلك أعقاب السجائر، هي أكثر أنواع النفايات التي يتم جمعها شيوعًا في جميع أنحاء العالم. وما يثير القلق بشكل خاص هو مرشح أسيتات السليلوز، وهو مادة مضافة بلاستيكية ضعيفة التحلل توجد بمعظم السجائر المصنعة تجاريًا. تم تقديم هذا المرشح من خلال صناعة التبغ لتقليل إدراك المدخنين للضرر والمخاطر، ولكن ليس له أي فائدة صحية. للتوعية بالسياسات والممارسات الصحية، وتحسين نتائج الصحة العامة، يمكن للحكومات والمجتمع الاستفادة من تقديرات التكلفة للوقاية من نفايات منتجات التبغ، و/أو التخلص منها، و/أو تنظيفها بشكل صحيح. إن تقدير تكاليف نفايات منتجات التبغ على المجتمعات والسلطات المسؤولة، يمكن أن يشجع على تطوير تدخلات السياسة الصحية والبيئية والمالية، وتحويل المساءلة عن تكاليف نفايات منتجات التبغ إلى صناعة التبغ العالمية. لدعم وضع السياسات الصحية والبيئية، فإننا نقترح بالتالي أسلوبًا تجريبيًا لتقدير التكاليف الاقتصادية لنفايات منتجات التبغ بناءً على العوامل الخارجية البيئية السلبية. نحن نقدم أولاً تقديرات عامة لست دول تمثيلية، ثم نحدد فجوات البيانات التي تحتاج إلى معالجة لوضع تقديرات عالمية. قد تكون التدخلات ضد نفايات منتجات التبغ قنوات جديدة لتنظيم منتجات التبغ عبر القطاعات، على سبيل المثال الصحة والبيئة والتمويل، وبالتالي تقليل الاستخدام العام للتبغ.

## 摘要

因吸烟导致的全球过早死亡率和致病率仍然很高。在每年全球售出的约 6 万亿支香烟中，大部分都被不当丢弃，成为污染环境的有毒废弃物。烟草制品废弃物（包括烟蒂）是全球最常见的废弃物。特别值得关注的是醋酸纤维滤嘴，它是一种难以降解的塑料添加剂，而大部分商业制造的香烟都使用这种滤嘴。烟草行业引入这种滤嘴，目的是让吸烟者认为降低了吸烟的危害和风险，但实际上它对健康并没有好处。为了普及卫生政策并关注和改善公共卫生状况，政府和社会可以通过在防止产生、适当处理和/或清理烟草制品废弃物方面进行成本估算来实现目标。估算烟草制品废弃物对社区和有关当局造成的经济成本，可以推动卫生、环境和财政政策相关干预措施的制定，并将处理烟草制品废弃物的成本转由全球烟草业承担。因此，为了支持卫生和环境政策制定，我们提出了一种实证研究法，根据烟草制品废弃物对环境的负面影响来估计其造成的经济成本。我们首先对六个代表性国家进行整体估计，然后确定需要解决的数据差距，以便估算全球经济成本。对烟草制品废弃物采取干预措施可能是跨部门（例如，卫生、环境和财政部门）管理烟草制品并从整体上减少烟草使用量的新渠道。

## Резюме

Курение табака по-прежнему является причиной значительной преждевременной смертности и заболеваемости во всем мире. Большая часть из примерно шести триллионов сигарет, продаваемых во всем мире каждый год, выбрасывается ненадлежащим образом и является токсичными отходами в окружающей среде. Отходы табачных изделий, в том числе окурки, являются наиболее часто собираемыми отходами во всем мире. Особую озабоченность вызывает фильтр из ацетата целлюлозы, который представляет собой плохо разлагаемую пластиковую добавку, входящую в состав большинства сигарет коммерческого производства. Этот фильтр предложен табачной промышленностью с целью уменьшить восприятие курильщиками вреда и риска, однако он не оказывает положительного влияния на здоровье. Предварительная оценка затрат на предотвращение, надлежащее удаление и (или) очистку отходов табачных изделий может быть полезной для правительств и общественности, так как позволяет придерживаться более информированных политик и практик в области здравоохранения, а также может улучшить результаты в сфере общественного здравоохранения. Оценка затрат, связанных с отходами табачных изделий, для сообществ и ответственных органов может способствовать разработке мер в области здравоохранения, охраны окружающей среды и налогово-бюджетной политики, а также переложить ответственность за издержки на отходы табачных изделий на мировую табачную промышленность. Поэтому для поддержки разработки политики в области здравоохранения и окружающей среды авторы предлагают эмпирический подход для оценки экономических издержек, связанных с отходами табачных изделий, на основе их отрицательных внешних воздействий на окружающую среду. Сначала авторы представят общие оценки для шести репрезентативных стран, а затем выявят пробелы в данных, которые необходимо устранить для разработки глобальных оценок. Мероприятия по борьбе с отходами табачных изделий могут стать новыми каналами регулирования табачных изделий в различных секторах (например, в сфере здравоохранения, окружающей среды и финансов) и, следовательно, сократить общее потребление табака.

## Introduction

Tobacco smoking contributes greatly to premature mortality and morbidity for millions of people worldwide every year. Tobacco product waste, including cigarette butts, smokeless products and cigar remnants, packaging, and the refuse associated with electronic nicotine and non-nicotine delivery systems and heated tobacco products, presents further health, environmental and economic concern for communities, provinces and countries.^1^ Local governments, private businesses and volunteers typically conduct clean-ups of tobacco product waste. Even with these vigorous efforts, tobacco product waste ends up on streets, sidewalks, beaches, parks, planters and parking lots; it flows through storm drains and streams to contaminate aquatic environments.

Self-reported data from smokers suggest that almost three quarters of them have discarded butts improperly.^2^ In New Zealand, 77% (168/219) of smokers were observed littering with their cigarette butts rather than disposing of them properly.^3^

Almost all the six trillion cigarettes sold globally have the poorly degradable cellulose acetate filter, and 20 of these filters weigh 3.4 g;^4^ therefore the annual global weight of discarded cigarette filters alone – assuming four trillion discarded improperly – is roughly 680 000 metric tonnes. Estimating the economic costs of preventing and reducing this tobacco product waste can inform health and environmental policy and practice.

There is a cost to society for preventing, properly disposing of, or cleaning up tobacco product waste. Several studies have documented these costs at the country or subnational level (Table 1). Using direct measurement methods, annual tobacco product waste costs were estimated to be 100 million United States dollars (US$; 2022 rate) in France^5^ and US$ 55 million in the United Kingdom of Great Britain and Northern Ireland.^6^ Using proportional estimation approaches, annual tobacco product waste costs were US$ 265 million collectively in the 30 largest cities in the United States of America^7^ and US$ 6 million in San Francisco, United States.^8^ Given the scope of sales and use of cigarettes and electronic nicotine and non-nicotine delivery systems worldwide, the economic burden of tobacco product waste is likely to be substantial for both low- and middle-income countries as well as high-income countries.

**Table 1:** Studies on costs of tobacco product waste globally

Country (location), year	Approach	Tobacco product waste cost estimate, million US$
France, 2021^5^	Direct measurement	93
United Kingdom, 2021^6^	Direct measurement	55
United States (30 of the largest cities), 2020^7^	Proportional estimation	265
United States (San Francisco), 2011^8^	Proportional estimation	6

US$: United States dollars.

Improper disposal of tobacco product waste in the environment is a negative externality (that is, a harmful effect to a third party, not directly involved in the matter, for which they are not compensated) borne by communities, governments and voluntary groups. The tobacco industry is ultimately the cause of this waste, but it emphasizes only downstream remedies for tobacco product waste by advocating smoker responsibility and by supporting clean-up and recycling efforts for cigarette butts.^1^

Aside from being a public nuisance, tobacco product waste may leach out harmful chemicals, including nicotine, metals and other toxic products,^9^^–^^11^ many of which are known human carcinogens.^12^ Tobacco product waste alters environmental quality, dirtying beaches, damaging public lands and degrading neighbourhoods with uncollected waste. Nicotine contaminates drinking water sources at levels above predicted so-called no-effect concentrations.^13^ Finally, discarded cigarette butts are a serious fire hazard for buildings and natural environments.

Estimating the environmental economic costs of tobacco product waste may lead to implementation of environmental and health policies that can help reduce tobacco use and its health harms. For example, mitigation fees could recover some costs of administering and implementing tobacco product waste clean-up programmes.^8^ These fees, which can increase over time, will raise the price of tobacco products, leading to a reduction in demand for such products. Thus, higher tobacco prices will lead to less tobacco consumption. An estimated 10% increase in the relative income price (defined as the percentage of per capita gross domestic product required to purchase 100 packs of cigarettes, using the lowest price from the Economist Intelligence Unit database) will reduce per capita cigarette consumption by 10% in high-income countries and less than 2% in low- and middle-income countries.^14^ These reductions may appear modest but when applied to millions of smokers worldwide, they will have substantial population health benefits.

Environmental economic policies can shift the responsibility of tobacco product waste prevention and mitigation from the consumer to the tobacco industry, so-called extended producer responsibility. Under this principle, industry must pay for the disposal and adverse impact of the hazardous waste its products create.^1^^,^^15^ This strategy may also counter the tobacco companies’ claims of their corporate social responsibility in imparting positive benefits on the environment, consumers, workers and society.^16^^,^^17^ Forcing this polluting industry to assimilate the true environmental and ecosystem costs of smoking will help reduce tobacco use. Other policy interventions may include deposit and take-back programmes for electronic nicotine and non-nicotine delivery systems, prohibiting the sale of filtered cigarettes and prohibiting smoking in public outdoor and indoor places.^18^ Estimating tobacco-related environmental economic costs can also be used to illustrate the economic benefits of reducing tobacco use.^19^

This paper focuses on the cigarette butt component of tobacco product waste, as currently available data only allow a defensible estimate of the economic burden of this particular category of tobacco product waste. Furthermore, we only describe methods for costing environmental externalities of tobacco product waste; methods for costing its health externalities can be added to this model, for example, when environmental^20^ and occupational^21^ comparative risk assessments can quantify the human health losses that may result from exposure to tobacco product waste. First, we review cost models for environmental pollution other than tobacco product waste. Next, we evaluate data sources needed for a tobacco product waste cost model at the subnational or national level; we then present a range of results for six sample countries in World Health Organization (WHO) regions. Finally, we identify data gaps in modelling the costs of prevention, clean-up, information, enforcement and other approaches to reduce tobacco product waste.

## Previous environmental waste cost models

The three main environmental concerns for tobacco product waste are water and soil pollution, and plastic waste; therefore, we sought similar examples of environmental contamination to conceptualize an approach for tobacco product waste. Two key concerns related to the cost of tobacco product waste are leaching of harmful chemicals into soil and water, even with proper landfill disposal, and community resources required for clean-up and disposal.^22^

Superfund sites in the USA are closed or abandoned high-risk hazardous waste sites, which endanger public welfare or the environment. Superfund sites are heavily contaminated with multiple pollutants, which can contaminate land, air and water. Tobacco product waste contaminates land and water, but we may also include costs associated with bioremediation, containment, excavation and incineration of contaminated solids (designated as direct costs) as well as the costs related to human health, environmental pollution and environmental injustice – that is, where certain groups, such as poor people or minorities, are disproportionally affected by exposure to pollution – for nearby communities (designated as secondary or indirect costs).^23^

### Benefit models

Society benefits when hazardous waste is prevented, remediated or mitigated. These benefits are challenging to quantify because there is no real market for hazardous waste clean-ups to determine a willingness to pay for such benefits. Willingness to pay is the maximum amount a stakeholder would pay for environmental goods or services including unspoiled and clean environments. Willingness to pay can, however, be inferred from choices people make in relation to cleaner environments. Revealed preference methods can be used to estimate willingness to pay by observing choices made by affected members of the community or society with respect to improving environments. The social damage caused by tobacco product waste is not equivalent to the level of damage caused by a United States superfund site, but we may be able to employ the same revealed preference methods to value the benefits of prevention, remediation or mitigation of tobacco product waste.^24^

### Single-use plastic waste cost models

Next, we considered cost models for plastic waste pollution, given that discarded cigarette filters are single-use plastics. We reviewed cost models for marine ecosystem losses, source reduction programmes and plastic environmental clean-up costs. However, we still do not know the human health impacts of environmental and occupational exposures to microplastics, and specifically those of tobacco product waste, because official health risk assessments and burden of disease estimates are not yet available.^20^^,^^21^^,^^25^

Over 300 million metric tonnes of plastic materials are produced globally each year, with an estimated half of this total discarded after single use and more than 40% ineffectively treated, for example, littered or disposed of in landfills.^26^^,^^27^ Single-use plastics account for an estimated 60% to 95% of all litter in the marine environment, equivalent to about 8 million metric tonnes annually.^28^^,^^29^ Although current estimates of the proportion of plastic litter attributable to tobacco products are not available, studies have estimated tobacco product waste to account for more than one fifth of all litter collected in cities and at beaches.^8^ However, this estimate does not account for the fact that cigarette filters ultimately break down into microplastics which are not included in litter estimates but may have a considerable impact on human and ecosystem health.

Direct costs resulting from single-use plastic waste can be estimated from the cost of collecting post-consumer plastic waste. A comprehensive cost model proposed for municipal waste collection includes fixed and variable costs per collection vehicle, and labour and personnel, container and carbon dioxide emissions costs based on the societal cost of carbon.^30^ Other models have focused either on labour costs associated with litter reduction or on specific waste streams, for example, single-use plastics.

Secondary costs resulting from single-use plastic waste are more challenging to quantify as plastic waste can have a wide range of impacts. Marine life may ingest plastic waste, which adversely affects ecosystems and human health, and marine litter may pollute tourism sites. These costs, which affect a particular industry or economic activity, can potentially be ascertained using market measures. Litter may also have non-market impacts, including the value placed on the marine environment.

## Estimating direct costs 

Conceptually, direct costs are associated with managing the problem (prevention and reduction), while negative costs, or benefits, are associated with the outcomes of mitigating tobacco product waste. We describe three basic methods to estimate the costs of tobacco product waste prevention and reduction and then identify data that can be used in these estimation methods.

### Methods

We start with the direct approach to measuring the cost of tobacco product waste prevention and reduction (*c(TPW)*), which includes both operating (for example, wages) and fixed costs (for example, capital). We could assess *c(TPW)* through surveys of public works, parks and recreational areas and other public administrative agencies. In low- and middle-income countries, however, voluntary groups rather than government departments would likely conduct such activities; thus, costs may not be captured in public databases, but their equivalent costs could be estimated using occupational wage data; see third approach. The limitation of this approach is that administrative agencies at the city, state or country level are unlikely to track or collect specific tobacco product waste data. It is, however, possible that these agencies would track data on general litter.

For the second (proportional) estimation approach, assuming one can obtain cost estimates for prevention and reduction of general litter, we could apply a weight to reflect the proportion of all collected litter, or all managed waste that is tobacco product waste. This proportion should be based on volume rather than weight, given that tobacco product waste is lighter in weight than other types of litter. We employed this method in previous studies,^7^^,^^8^ including using litter survey data and overall litter reduction costs for the city of San Francisco, California and the World Bank review of solid waste management.^31^ The basic model for cost estimation is: *c(TPW) = λc(all)* where *c(TPW)* is the cost of tobacco product waste prevention and reduction, *λ* is the percentage of all litter attributable to tobacco product waste and *c(all)* is the estimated costs of the prevention and reduction of all litter. Thus, the more accurate the measure of *c(all)* and the more precisely we measure λ, the better the estimate of *c(TPW)*.

The third approach uses occupational wage data, shared by national statistics offices with the International Labour Organization (ILO)^25^ to estimate the *c(all)*. This approach is particularly useful when litter prevention and reduction costs are unknown or are highly uncertain, but it is less comprehensive since it omits fixed costs. In these cases, it may be possible to estimate *c(all)* with country data reported to ILO, which in turn can be used to estimate *c(TPW)* by applying the tobacco product waste proportion (*λ*). We borrowed this job-cost matrix approach from the job-exposure matrix used in occupational epidemiology, where a certain job is considered a proxy for a certain level of exposure to an occupational risk factor.^32^ This approach may be feasible at national levels for all countries, with potential ultimately for global estimation.

### Data sources

Several data elements are associated with the prevention and reduction of tobacco product waste, and these data could be collected specifically for tobacco product waste or for general litter more broadly (Box 1). Cities and countries will vary considerably in these expenditures, and for many, some elements may not be relevant. Models for tobacco product waste prevention and reduction can be developed with options for including or excluding certain data elements.

Box 1 **Data sources for estimating the direct costs of tobacco product waste, by cost areas^a^**

- Implementation of litter regulations, for example, signs, education and administration^33^

Potential data source^b^: marine litter legislation^34^

- Litter prevention, for example, regulation enforcement and courts^33^

Potential data source^b^: marine litter legislation^34^

- Mechanical street sweeping^c^

Potential data source: government – labour and equipment^33^^,^^35^

- Manual street and sidewalk cleaning^c,d^

Potential data source: government – labour and equipment^33^

- Manual street and sidewalk cleaning^c,d^

Potential data source: private – labour costs^33^

- Manual area clean-up, for example, parks, beaches and bodies of water^c^

Potential data source: government – labour and equipment^33^

- Stormwater systems clean out^c^

Potential data source: government – labour and equipment^33^^,^^36^

- Stormwater and wastewater treatment^c^

Potential data source: government – labour and filter cleaning^33^

- Landfill (weight-based fees)^37^

Potential data source^b^: global snapshot of solid waste management to 2050^38^

- Aggregate measures of direct litter costs, that is, not directly specific to tobacco product waste^33^

Potential data sources^b^: marine litter^34^^,^^39^

- Hazardous waste management (electronic nicotine and non-nicotine delivery systems and heated tobacco products)^31^

Potential data source^b^: e-cigarette waste^40^

^a^ Includes examples of known United States data sources.^b^ Includes sources with direct estimates and sources with components of cost estimates.^c^ Limited data are available globally; these costs must therefore be estimated individually for cities and countries.^d^ Manual street and sidewalk cleaning operated by the government is assumed to include a mix of labour and equipment costs, whereas such cleaning by private industry is assumed to be mainly labour costs.

Implementation of the second and third approaches described in the previous section requires estimates of additional parameters, including population, smoking prevalence, tobacco sales and proportion of tobacco products that are littered. It is also useful to have an estimate of the percentage of all litter or tobacco product waste that is expected to be reduced (*α*), which can be applied as a weight in the second and third estimation approaches. The percentage of litter attributable to tobacco product waste can also be estimated from general clean-ups sponsored by voluntary groups.

Additional data are needed for the third estimation approach, where *c(all)* is unknown and we use a job-cost matrix approach (Box 2). Only workers in tobacco product waste management and reduction are included, and estimates of monthly wages and number of workers by the International Standard Classification of Occupations code (to two-digit level) and country are available from the ILO. Estimates of the percentage of occupations related to tobacco product waste engaged in tobacco product waste management and reduction (*β*) and the percentage of tobacco product waste-mitigation activities carried out by management versus labour (*ϕ*) are needed.

Box 2 **Data needed for proportional prevention and reduction cost models for tobacco product waste**

- Population: total and annual visitors^41^

Potential data sources include: Eurostat data sets and reports^42^, World Tourism Barometer^43^ and World Bank population estimates and projections^44^

- Smoking prevalence^45^

Potential data sources include: WHO global report^46^

- Tobacco product sales^47^

Potential data sources include: WHO global report^46^ and the Maxwell report^48^

- Percentage of tobacco products littered^33^
- Percentage of all litter expected to be reduced (*α*)^a,b^
- Percentage of all litter attributable to tobacco product waste (*λ*)^33^^,b^
- Monthly wages in occupations related to tobacco product waste management and reduction (from the ILO, by ISCO code, by country)^25^
- Total workers in occupations related to tobacco product waste management and reduction (from the ILO, by ISCO code, by country)^25^
- Percentage of workers in occupations related to tobacco product waste management and reduction engaged in tobacco product waste management and reduction (*β*)^a,b,c^
- Percentage of mitigation activities for tobacco product waste carried out by management versus labour (*ϕ*)^a,b^
- Estimated share of ISCO occupations related to tobacco product waste (*δ*)^a,b^

ILO: International Labour Organization; ISCO: International Standard Classification of Occupations.^a^ Parameter estimate may be necessary if it is not feasible to measure the direct effect.^b^ Can be estimated or extrapolated using geographically limited data.^c^ Expected to vary by type of occupation.

We applied the proportional estimation (the second approach) to estimate costs in six representative countries across WHO regions (Table 2). Annual tobacco product waste costs range from about US$ 50 million in Jordan to more than US$ 2.2 billion in China. Since these are limited to direct costs only, they should be considered an underestimate of overall annual tobacco product waste costs for these selected countries. Secondary costs are not included in these estimates.

**Table 2:** Cost estimates of tobacco product waste management and reduction in one country in each of the six WHO regions, 2021

Country	Smoking prevalence^a^, %	Estimated global tobacco product waste, %	Estimated costs of all product waste^b^, US$	Estimated tobacco product waste^c^, %	Estimated costs of tobacco product waste^d^, US$
Brazil	12.80	NA	1 323 319 752.49	13.43	177 725 562
China	23.50	NA	9 299 458 020.90	24.66	2 292 981 931
Germany	22.00	NA	891 774 234.17	23.08	205 850 882
India	8.00	NA	8 000 741 085.82	8.39	671 576 259
Jordan	34.80	NA	137 045 667.78	36.51	50 040 275
South Africa	20.30	NA	482 288 285.88	21.30	102 725 410
**Global**	**22.30**	**23.40^e^**	**NA**	**NA**	**NA**

NA: not applicable; US$: United States dollars; WHO: World Health Organization.

^a^ Source: WHO global report on trends in prevalence of tobacco use 2000–2025.^49^

^b^ Based on published literature and reports. For Brazil, China and India, we were not able to identify public sources of data on the costs of all product waste; therefore, for these countries, we imputed a cost of all product waste per capita based on an average of other middle-income countries (US$ 7.85 per capita).

^c^ Based on the global average of data compiled annually as part of the Ocean Conservancy International Coastal Cleanup and calculated as the percentage of all product waste that is tobacco product waste and weighted by WHO estimates of smoking prevalence in each country, that is, we assumed that countries with higher rates of smoking would have higher proportions of tobacco product waste. The tobacco product waste percentage for country(*i*) was calculated as: weight(*i*) × mean global tobacco product waste percentage, where weight(*i*) = smoking prevalence(*i*)/mean global smoking prevalence.

^d^ Based on multiplying all product waste cost by the tobacco product waste proportion, using unrounded significant digits. These figures do not include any costs associated with voluntary non-commercial waste pickers.

^e^ The numerator and denominator for the percentage are 964 521 collected waste items and 4 122 225 collected waste items, respectively.

## Modelling secondary costs

Secondary costs of tobacco product waste include tangible losses, such as lower business revenues, and intangible losses, such as lower resource values caused by blight (Box 3). What society is willing to pay to avoid tobacco product waste in urban areas, on beaches, in aquatic habitats and in other natural environments are considered secondary costs. Secondary effects of tobacco product waste pollution are associated with the environmental concept of ecosystem services preservation. Ecosystem services include: food supply; regulating services, for example, water and waste purification; and cultural and aesthetic services including tourism and recreation.

Box 3 **Willingness-to-pay variables for modelling secondary costs of tobacco product waste and data sources**

**Beaches free of tobacco product waste**

Key informant surveys, for example, businesses, tourist agencies, lifeguards and environmental groups

Population representative surveys

**Urban streets and sidewalks free of tobacco product waste**

Key informant surveys, for example, city government officials, businesses, neighbourhood groups, trash management authorities and advocates

Population representative surveys

**Unpolluted aquatic biomes, free of not just macro trash, but also of chemical toxins and microplastic contaminants**

Key informant surveys, for example, water boards, environmental groups and ocean protection authorities

**Tobacco-free societies**

Key informant surveys, for example, government officials and tobacco control professionals

Population representative surveys

Tobacco product waste pollution deprives people of clean natural environments, but tobacco product waste also degrades urban environments. Single-use plastics, including discarded cigarette filters, can be transported into rivers, streams and oceans, thus linking urban tobacco product waste to secondary ecosystem losses. The question is: what costs are citizens willing to pay to preserve ecosystem services and social conditions that are affected by tobacco product waste? In addition, quantitative data based on secondary losses suffered by businesses, tourism and property owners due to fires attributable to cigarettes, decreased asset valuations and the public nuisance of tobacco product waste should be included in a total cost model.

Combining direct and secondary costs will provide an estimate of the costs of tobacco product waste beyond prevention, mitigation and clean-up to a broader concept of ecosystem damages. Given the length of time tobacco product waste has been accumulating in the environment through inappropriate disposal of the trillions of mostly filtered cigarettes smoked each year, these costs are likely to be high. However, for this strategy to give a feasible estimation globally, a detailed database as outlined is necessary, and this will be a longer-term undertaking.

## Conclusions

We aimed to develop a method to model direct and secondary environmental economic costs of tobacco product waste. Because direct cost data are more readily obtained from local sources, it may be most appropriate to focus initially on the city, county, state or provincial level. The best approach would be to complete multiple local studies and then extrapolate the estimates from these studies to national, and ultimately global, levels. Data sources available in high-income and low- and middle-income countries will vary considerably, but the approach to quantifying the environmental costs of tobacco product waste relies on economic modelling and not on precise data from each location. Article 5.3 of the WHO Framework Convention on Tobacco Control requires the tobacco industry to provide information to governments that could be useful in this modelling exercise. However, it is also important to recognize that such data must be critically evaluated as the industry is not a legitimate stakeholder in this process and has a history of providing misinformation and creating confusion about the environmental impacts of tobacco products.^50^

The direct costs of tobacco product waste may be extrapolated from tobacco product marketing data (cigarette sales and consumption at the local level), proportional estimates of tobacco product waste in the entire waste stream, and mitigation costs based on labour costs, disposal costs, programme administrative costs and the cost of other components of tobacco product waste management. Implementation of this approach may be possible in the short-to-medium term with available data, including for low- and middle-income countries.

The secondary costs of tobacco product waste may be derived from business loss estimates, the environmental impact of fires caused by cigarettes and willingness to pay estimates based on established economic models including the hedonic price model, where stakeholders may assign a price to achieving an environment free of tobacco product waste. Since this approach is less developed in previous models and lacks available data, it may only be feasible with substantial additional efforts, including new data collection across both low- and middle-income countries and high-income countries

Given that we still do not know the potential health impact of environmental and occupational exposures to microplastics in general and those of tobacco product waste, research is needed to close this data gap. Facilitating risk assessment of microplastic exposure can inform policy development to better regulate this potentially harmful exposure. However, lack of understanding does not negate the urgent need for government policy and action to prevent potentially harmful exposures nor the ability of such policies to reduce exposure. Early action is critical because the cost of cleaning up or responding to pollution generally requires greater time and resources than preventing the exposures in the first place. Better data can inform most effective interventions to limit exposures and resulting health outcomes. However, given that these exposures are often ongoing and ubiquitous and they occur in conjunction with numerous other environmental contaminants, early and effective action to reduce exposures in general will likely have a significant impact on protecting the public’s health.
